# Ethical issues in research involving minority populations: the process and outcomes of protocol review by the Ethics Committee of the Faculty of Tropical Medicine, Mahidol University, Thailand

**DOI:** 10.1186/1472-6939-14-33

**Published:** 2013-09-11

**Authors:** Pornpimon Adams, Waranya Wongwit, Krisana Pengsaa, Srisin Khusmith, Wijitr Fungladda, Warissara Chaiyaphan, Chanthima Limphattharacharoen, Sukanya Prakobtham, Jaranit Kaewkungwal

**Affiliations:** 1 Office of Research Services, Faculty of Tropical Medicine, Mahidol University, Bangkok, Thailand; 2 Department of Social and Environmental Medicine, Faculty of Tropical Medicine, Mahidol University, Bangkok, Thailand; 3 Department of Tropical Pediatrics, Faculty of Tropical Medicine, Mahidol University, Bangkok, Thailand; 4 Department of Microbiology and Immunology, Faculty of Tropical Medicine, Mahidol University, Bangkok, Thailand; 5 Department of Tropical Hygiene, Faculty of Tropical Medicine, Mahidol University, Bangkok, Thailand

**Keywords:** Ethical considerations, Minority groups, Vulnerable populations, Research proposal

## Abstract

**Background:**

Recruiting minorities into research studies requires special attention, particularly when studies involve “extra-vulnerable” participants with multiple vulnerabilities, e.g., pregnant women, the fetuses/neonates of ethnic minorities, children in refugee camps, or cross-border migrants. This study retrospectively analyzed submissions to the Ethics Committee of the Faculty of Tropical Medicine (FTM-EC) in Thailand. Issues related to the process and outcomes of proposal review, and the main issues for which clarification/revision were requested on studies, are discussed extensively.

**Methods:**

The study data were extracted from proposals and amendments submitted to the FTM-EC during the period October 2009 – September 2012, and then analyzed qualitatively and quantitatively. The main issues for clarification/revision were analyzed by thematic content analysis.

**Results:**

373 proposals were submitted; 44 studies involved minority groups with 21 extra-vulnerable minorities. All clinical and 2/3 of non-clinical studies submitted for initial review underwent full-board review. For combined clinical and non-clinical study submissions, 92.1% were referred back to the investigators and approved after clarification/revision, while 2.7% were deferred due to major/critical changes, and 2.1% not approved due to substantial violations of ethical principles. The main issues needing clarification/revision differed between all studies and those involving minorities: participant information sheet (62.2% vs. 86.4%), informed consent/assent form (51.2% vs. 86.4%), and research methodology (80.7% vs. 84.1%), respectively. The main ethical issues arising during the meetings, regarding studies involving minorities, included ensuring no exploitation, coercion, or pressure on the minority to participate; methodology not affecting their legal status; considering ethnicity and cultural structure; and providing appropriate compensation.

**Conclusion:**

Delays in the approval or non-approval of studies involving minorities were mainly due to major or minor deviations from acceptable ethical standards and/or unclear research methodology. The FTM-EC has employed several mechanisms in its operations, including transparency in the review process, building good relationships via open communication with investigators, requesting investigators to consider closely the necessity to enroll minority groups and the risk-benefits for individuals and their communities, and the inclusion of minority-community engagement when developing the proposal. Other effective activities include annual study-site inspections, and offering refresher courses to raise awareness of minority and vulnerability issues among researchers.

## Background

Institutional review boards (IRBs) typically apply common rules and regulations to protect human research subjects, in accordance with Title 45 § 46 of the US Code of Federal Regulations (45 C.F.R. § 46) regarding informed consent process, balance of risks and benefits, protection of participant privacy, and other requirements for the approval of proposed research. Additional subparts of 45 C.F.R. § 46 are also usually enforced for the specific protection of certain vulnerable populations, including pregnant women, fetuses, and neonates (Subpart B); prisoners (Subpart C); and children (Subpart D) [1]. According to the Council for International Organizations of Medical Sciences (CIOMS) guidelines of 2002, vulnerable research participants should receive special attention, as they may suffer from stigmatization, have limited power, lower educational levels, poverty, limited resources, inadequate physical strength and/or other necessary attributes to protect and defend their own interests [2]. IRBs thus play major roles in safeguarding against the potential coercion, inducement, and exploitation of these particularly vulnerable groups [3-5].

For project proposals, the most common set of secular principles employed by IRBs include respect for autonomy, non-maleficence, beneficence, and justice [2]. A key responsibility of IRBs is to balance moral and humane principles with the likelihood of gaining new knowledge from the conduct of research involving humans, which could benefit society in general. However, in attempting to safeguard ethical standards, IRBs have obligations to all stakeholders. They have obligations to ensure and protect the rights of study participants, to the society that provides the resources for research, and to researchers, and the obligation to treat their proposals with just consideration and respect [6]. The management of these principles, roles, and obligations can be highly complex, and conflict may arise since they are not necessarily complementary or mutually exclusive. It may be especially sensitive when research studies involve more vulnerable populations. Special attention is usually required when study populations are incompetent persons, women (pregnant or not), children, prisoners, refugees, other socio-economically disadvantaged groups, and ethnic minority groups [4,5,7]. It becomes even more complex, usually requiring extensive discussion among IRB members, when studies involve participants with multiple vulnerabilities, described herein as “extra-vulnerable” populations, e.g., pregnant women or the fetuses/neonates of ethnic minorities, children inhabiting refugee camps, or illegal cross-border migrants.

This study retrospectively analyzed all study applications submitted to an IRB, namely the Ethics Committee of the Faculty of Tropical Medicine (FTM-EC), Mahidol University, Thailand. The FTM-EC adopts and complies with international standards in the review of protocols involving human research participants; its review process includes individual consideration and group discussion of the relevant issues. Discussion before reaching consensus ensures that the basic rights of human-research subjects are protected. Corresponding to the standard ICH and CIOMS guidelines [2,8] and the specific ethical guidelines proposed for minority populations [7,9,10], the FTM-EC members consider the core ethical principles, as follows: (a) do no harm to the individual, (b) respect the privacy of participants; (c) maintain the confidential information and anonymity of participants (which is becoming critical, especially when a study employs peer groups and interpreters in minority ethnic communities), and (d) assure an appropriate informed-consent process (with special attention to the literal translation of the “Participant Information Sheet” in the language understood and preferred by minority ethnic participants).

The FTM-EC has extensive experience of studies involving vulnerable and extra-vulnerable populations in Thailand. Besides core ethical principles, special concerns about exploiting vulnerable and extra-vulnerable study participants have routinely and extensively been expressed by FTM-EC members. According to the CIOMS guidelines [2], any study requiring the recruitment of vulnerable research subjects must strictly apply “special justification”, such that: (a) it is scientifically necessary to conduct the study with a vulnerable subject group; (b) the study is intended to obtain knowledge and assurance that the results could improve diagnosis, prevention or treatment for the vulnerable group; and (c) the risks for the vulnerable group would not exceed those for other groups.

The main objective of this study was to review and analyze the FTM-EC research proposal review process and outcomes, with particular attention to minority populations. Other studies exploring the ethical review process suggested that study investigators were typically requested to clarify the proposal’s informed consent process; other issues included failure to comply with good clinical practice, lack of adequate information, and discrepancies in the information provided [11,12]. This study also examined the main issues for which clarification/revision were requested, particularly from initial and continuing reviews of studies involving minority groups, and deferred or non-approved studies.

## Methods

The study data were collected from all proposals and amendments submitted to the FTM-EC for initial and continuing review during the period October 2009 – September 2012. FTM-EC has been functioning since 1993 and it is one of ten IRBs officially recognized by the Food and Drug Administration (FDA) of Thailand. Since 2009, two Ethics Committee panels have been formed: Panel 1 mainly reviews clinical studies, while Panel 2 reviews non-clinical trials. The functions of the two panels differ in terms of proposal review. Panel 1 is composed of 18 members, 9 physicians, 7 non-physicians (medical technology, research nurses, statisticians, lawyer, research management/operational staff) and 3 laypersons; this panel reviews protocols that are classified as clinical trials/ research. Panel 2, composed of 13 members, 3 physicians, 8 non-physicians and 3 laypersons (the lawyer also acts as a lay person), reviews all non-clinical research, including biomedical science (for both laboratory and field studies), social science, observational and epidemiological research, and postgraduate-student research studies. Both panels perform under the same standard operating procedures (SOPs) in their monthly review of research proposals.

Panel 1 convenes during week 1, and Panel 2 during week 2 of each month. They review both initial and continuing studies. “Initial review” refers to the first submission of a protocol to the FTM-EC for review, whereas “continuing review” includes studies where the amendment of a protocol or continual annual approval has been requested. Continuing review requires full-board review when the protocol amendment includes major issues, e.g., changes in sample size, drug dosage, or amount of blood drawn. Regarding the request for annual approval review, researchers are required to report study progress and any serious incidents occurring during the conduct of the study for FTM-EC review; thus, the primary committee members overseeing the request can detect any change or deviation/violation from the original proposal. Annual reviews are submitted together with amendments, if any change(s) are proposed to the original protocol. According to the FTM-EC guideline for researchers, “amendment” is defined as a written description of a change(s) to or formal clarification of a protocol. Staff of the Ethics Committee Secretariat, Office of Research Services, Faculty of Tropical Medicine, collect all comments from the (typically) two primary reviewers, and all other Ethics Committee members prior to the meeting, and also record all discussions during the meeting. Issues raised by all reviewers are then summarized for the investigators who submitted the proposal. The average number of research project proposals reviewed per month was 10.36 (3.86 for Panel 1 and 6.5 for Panel 2). To protect the rights and welfare of human participants and ensure that the research projects approved by the committee are conducted according to the stated methodology, the FTM-EC has specified a policy of random annual site visits to selected study sites with ongoing projects, usually one clinical and one non-clinical study per year. According to its SOP, the Chairperson of each panel designates Ethics Committee members responsible for collecting and recording a non-compliance inventory. For the selected project, the investigators are informed ahead of time with the details of what will be inspected/monitored on the agreed-upon visit date. The monitoring team prepares a checklist, reviews the necessary documents (e.g., SAE and unexpected events reports) and scopes the site visit. A field briefing meeting of the monitoring team and the investigators is held before the actual site visit (the briefing covers the purpose of the visit and what is to be reviewed) and afterwards (what was observed and explanations/clarifications). A formal summary report is provided to the investigators after the monitoring activities have been completed.

During the study period, and with permission from the Chairpersons of both Panels 1 and 2, initial and amended proposals are examined for type (clinical or non-clinical) and study population (minority or otherwise). Proposals are also categorized into studies involving ethnic-minority populations, which include displaced populations, cross-border populations, refugees, and legal and illegal migrant workers in different settings. Of the minority populations identified, “extra-vulnerable minorities” refers to pregnant women, fetuses and neonates of ethnic minority populations.

The relevant study data were extracted from documents filed at the secure FTM-EC facility, then analyzed qualitatively and quantitatively by the research team, who are members of the two FTM-EC panels. Summary statistical data for the review process and outcomes were determined according to FTM-EC guidelines. Adopting international standards for protocol review, the FTM-EC Chairpersons and Committee Secretary classify each proposal into type for review, for allocation to the appropriate Committee. The criteria for full-board review, which is determined by the Chairperson, are studies that involve more than minimal risk and involve elements, procedures, or interventions that require additional provisions or safeguards. Expedited review is potentially available to proposals with minimal risk, which may include identifiers (direct or indirect), topics that are not sensitive, populations that may include regulated vulnerable populations and others with adequate protection, and studies that require continuing IRB review. Expedited review may also be granted for studies that have submitted annual reports where no serious adverse events have been noted. Exempt review includes studies with minimal risk, do not include identifiers, topics are generally not sensitive, involve non-vulnerable populations, are exempt from the formal informed-consent requirement, and are exempt from continuing IRB review.

Based on FTM-EC guidelines and the standard review form, Committee members decide on the review outcomes as follows: approval (affirmative decision that the submitted protocol may be conducted as per plan); approval after amendment (affirmative decision with the requirement that amendment(s) be incorporated, as recommended); approval with clarification (affirmative decision after satisfactory clarification(s) have been made), deferment (protocol is not recommended for approval as submitted, but can be re-assessed after revision); disapproval (protocol is not recommended for the reasons specified by the Committee). In this study, “approval after amendment” and “approval with clarification” were grouped together as protocols requiring clarification/revision.

The main issues for which clarification or revision were requested by the FTM-EC, as shown in the meeting reports, were grouped under selected themes, and subjected to thematic content analysis. The thematic content is based mainly on the ethical principles/issues presented on the standard FTM-EC review form, which accompanies the protocol provided to each FTM-EC member as a guideline for his/her review. The checklist on the FTM-EC review form includes issues of scientific merit, sampling techniques, investigators’ experience, adequacy of the research facilities, realistic budgeting, appropriate compensation, study population and vulnerable populations, type of study, information sheet, and informed consent process. Particular thematic points of concern regarding the protocol on the form also include, for example, project title, objectives, research methodology, protection of privacy and confidentiality, major ethical issues, participant information sheet, informed consent/assent form, questionnaire/case record form (including either a printed, optical, or electronic document designed to record all of the information required for the protocol), advertisement/notification about the study, investigator brochure, and materials transfer agreement (MTA). However, the FTM-EC may add matters of particular concern to the basic review form. The content analysis in this study was synthesized from all issues discussed and recorded during the monthly meeting, including, e.g., research design and study plan, recruitment method, informed-consent process, risk and benefits of the participants, confidentiality and security of the study data, and potential societal impact. If one or more statements addressed an issue related to any of the major themes, the item was tagged for content analysis. In the analysis of all documents archived at FTM-EC, 4 faculty members assessed and confirmed the thematic content. Reflecting the FTM-EC meeting format, consensus was achieved on various issues, and the opinions, conclusions, and recommendations reached were not those of the authors alone.

## Results

### Process of reviewing studies involving minority populations

During the 3-year study period, 96 clinical and 195 non-clinical studies were submitted for initial ethical review, and 43 clinical and 39 non-clinical studies for continuing review. Among these, 33 clinical and 11 non-clinical research studies involved minority groups in Thailand, including cross-border populations, displaced persons, and/or refugees, and legal and illegal foreign workers in urban areas of Thailand.

As Table 1 shows, 373 studies were submitted for ethical review; 291 for initial review and 82 for continuing review. Forty-four (44) studies involved minorities, 23 targeting generic minority populations, and 21 extra-vulnerable minority populations. Of 291 proposals submitted for initial review, 77.3% (225/291) underwent full-board review, 20.3% (59/291) expedited review, and 2.4% (7/291) were exempted from ethical review. To safeguard the rights, safety, and well-being of all trial participants, and in compliance with the responsibilities of IRBs outlined in the ICH Guideline for Good Clinical Practice [8], all clinical studies, including studies involving minority groups, submitted for initial review by the FTM-EC, underwent full-board review, and none was exempted. On the other hand, of the non-clinical studies submitted for initial review, only two thirds (66.1%) underwent full-board review (where the research protocol called for the collection of primary data); and about one third underwent either expedited review (including studies proposing the use of un-identified leftover specimens with official authorization or analysis of secondary data) or were exempted (including studies that used public data or information originally from non-research program evaluation projects). All 44 studies involving minority groups underwent full-board review. Regarding the continuing review of clinical studies, most (74.4%) underwent expedited review, since no major protocol amendment, nor amendment with greater than minimal additional risk, were involved.

**Table 1:** Ethical review outcomes of studies at FTM-EC, 2009-2012

**Ethical review process and outcomes**	**All studies**				**Studies involving minority groups**			
	**Clinical studies**		**Non-clinical studies**		**Clinical studies**		**Non-clinical studies**	
	**Initial review**	**Continuing review**	**Initial review**	**Continuing review**	**Without extra-vulnerable minority**	**With extra-vulnerable minority**	**Without extra-vulnerable minority**	**With extra-vulnerable minority**
	**N = 96**	**N = 43**	**N = 195**	**N = 39**	**N = 19**	**N = 14**	**N = 4**	**N = 7**
	**n (%)**	**n (%)**	**n (%)**	**n (%)**	**n (%)**	**n (%)**	**n (%)**	**n (%)**
**Type of review**								
**Full board**	96 (100%)	11 (25.6%)	129 (66.1%)	1 (2.6%)	19 (100%)	14 (100%)	4 (100%)	7 (100%)
**Expedited**	0	32 (74.4%)	59 (30.3%)	38 (97.4%)	0	0	0	0
**Exempted**	0	0	7 (3.6%)	0	0	0	0	0
**Initial review outcome**								
**Simple approval**	0	27 (62.8%)	13 (6.7%)	38 (97.4%)	0	0	0	0
**Approval after clarification/revision**	92 (96%)	13 (30.2%)	176 (90.2%)	0	17 (89.4%)	12 (86%)	3 (75%)	7 (100%)
**Deferment**	3 (3%)	0 (0%)	5 (2.6%)	0	1 (5.3%)	1 (7%)	0	0
**Non-approval**	1 (1%)	3 (7%)	1 (0.5%)	1 (2.6%)	1 (5.3%)	1 (7%)	1 (25%)	0

Notes: 1. “Continuing review” includes studies where an amended protocol was requested and continual annual approval was awarded.

2. “Extra-vulnerable minority” includes pregnant women, fetuses, and neonates.

3. Percentages in parentheses are relative to N of respective column.

### Outcomes of the reviews of studies involving minority populations

Regarding the results of initial review for combined clinical and non-clinical study submissions, 92.1% (268/291) were referred back to investigators and approved after satisfactory clarification and/or revision, while 2.7% (8/291) were deferred, and 2.1% (6/291) not approved. Of the initial FTM-EC review of clinical studies, none was awarded simple approval, while most continuing reviews (62.8%) were. In terms of initial review outcomes for non-clinical studies, only 6.7% of proposals received simple approval. A few initial review studies were deferred (3% of clinical and 2.6% of non-clinical studies) due to the Ethics Committee’s consensus that the study required major and critical changes. Similarly, a few initial studies were not approved (1% of clinical and 0.5% of non-clinical studies) and some continuing review studies were not approved (7% of clinical and 2.6% of non-clinical studies), since the proposed study or its amendments were considered in major violation of ethical principles.

Of the 44 studies involving minority groups undergoing full-board review, none was granted simple approval. Overall, 88.6% (39/44) were approved after clarification and/or revision, 4.5% (2/44) were deferred, and 6.8% (3/44) not approved. Among clinical studies involving minority populations, 5.3% without extra-vulnerable groups and 7% with extra-vulnerable populations were deferred; and the same rates were not approved. One non-clinical study involving a minority, but without extra-vulnerable status, was not approved.

### Reasons for protocol revision or deferral/non-approval

Proposals that were not granted simple approval underwent subsequent submissions, incorporating responses to FTM-EC suggestions. Among the clarifications/revisions requested, research methodology, participant information sheet, recruitment (inclusion–exclusion criteria), and informed consent/assent form, were among the most frequent issues. Table 2 shows the main issues of clarification/revision needed for re-submission; the top three reasons were participant information sheet (86.4%), informed consent/assent form (86.4%), and research methodology (84.1%). However, for all studies, research methodology was the issue most frequently requiring clarification/revision (80.7%), followed by participant information sheet (62.2%), and recruitment, specifically inclusion–exclusion criteria (60.1%). The common matters for which clarification and amendment were requested included inadequate information on the participant information sheet, the lack of lay terms to facilitate laypersons’ comprehension, contradictory information in the protocol, lack of approvals and signatures by authorized personnel, inadequate sample size, unclear definitions of inclusion/exclusion criteria, and poor hypotheses and research questions. The reasons for deferment and non-approval were also investigated, as shown in Table 3.

**Table 2:** Main issues for which clarification/revision were requested

**Main issues**	**Studies involving minority group**		**All studies**	
	**N = 44**		**N = 291**	
	**n**	**(%)**	**n**	**(%)**
Participant information sheet	38	86.4	181	62.2
Informed consent/assent form	38	86.4	149	51.2
Research methodology	37	84.1	235	80.7
Recruitment (inclusion–exclusion criteria)	32	72.7	175	60.1
Procedures for treatment and care	29	65.9	103	35.4
Risk-benefit considerations	27	61.4	108	37.1
Compensation	26	59.1	76	26.1
Sample size	21	47.7	122	41.9
Minor typographic errors	13	29.5	70	24.0
CRF/Questionnaire/MTA	8	18.2	128	43.9

**Table 3:** Main issues for which studies involving minority groups were deferred/not approved, 2009-2012

**Main issues**	**Studies involving minority groups**		**All studies**	
	**Deferment**	**Non-approval**	**Deferment**	**Non-approval**
	**n = 2**	**n = 3**	**n = 8**	**n = 6**
No scientific merit	1	2	1	4
Unsound research methodology	2	3	4	6
Risk-benefit concerns (individual and community)	1	2	3	4
Human rights violations	-	1	-	1
No FTM staff involvement and/or not implemented within FTM	-	-	1	-
Required ethical clearance from elsewhere (Ministry of Public Health), not from FTM-EC	-	-	1	1
Research conducted and completed before ethical clearance requested	-	-	1	-

Specific protections are focal at FTM-EC meetings when studies involve minority groups, particularly those enrolling under-represented minority populations, such that culturally sensitive values and the trust of minority ethnic communities are respected and maintained. The principles usually recorded in FTM-EC meeting reports for referral to investigators for clarification/revision include: (a) ensure no exploitation, coercion, or pressure to participate among the minority, which is regarded as a vulnerable population; (b) ensure that the research methodology does not affect the legal status of the minority; (c) consider the necessary educational level and competency of the minority research subjects to participate in the study; (d) understand and consider the ethnicity and cultural issues of the minority communities being studied; (e) ensure that the research methodology and instruments are accepted and understood by minority ethnic participants; and (f) provide appropriate compensation for the contributions of the minority participants residing in under-served and limited-resource environments.

After the FTM-EC had made its decision on a particular proposal, the reasons for clarification/revision or deferment/not approval were clearly explained to the investigators. Selected anonymized excerpts from correspondence between FTM-EC and investigators are presented as examples.

On the study objective:

FTM-EC comment - The objectives are not clear. For example, on page 4/10, the objective of the study is stated as “To determine the impact of treated XXX infection”; does this mean that the impact on fetal and newborn growth is the result of the treatment given to the mother? If this is the case, can pregnant women without XXX infection serve as a control group?

Investigator response: As per your suggestion, we have changed the objective to “To determine the impact of XXX infection and XXX treatment in pregnancy on fetal and newborn growth and relate these data to non-infected mothers and to international growth standards.

On the research methodology:

FTM-EC comment - For the pharmacokinetic study, is it possible to have the total blood volume taken from each infant?

Investigator response - After careful discussion with the hematology and pharmacokinetics labs, we were able to reduce the round total blood volume from XX ml to XX ml in pages 9 and 17 of the research proposal (exact total reduced from XXX to XXX). Further reductions do not seem possible without reducing the scientific value of the study. We hope you will find this acceptable.

FTM-EC comment - In #14.2 “as many women as possible will be recruited” is not acceptable. If so, when will the project end? Sample size calculation is needed. In fact, you have stated in the explanation form that XXX participants will be recruited. How did you determine the number XXX?

Investigator response - We plan to study a total of XXX women. This sample size will allow us to detect a difference of X.X% of the endpoint with 80% power and alpha = 0.025 between the two groups of women. Allowing 20% drop-out the total number of women to be recruited into the study is XXX.

On the informed-consent process:

FTM-EC comment – The participant information sheet (PIS) does not state the risk for patients who do not have the standard treatment. Please state this clearly in the PIS.

Investigator response - The PIS has been revised to clearly explain that XXX is not a standard treatment and explain the possible risk to patients on page 4 of the PIS Thai version and page 5 of the PIS English version.

FTM-EC comment – If this project recruits Thais only, then only a Thai version of the PIS is needed. If not, please provide the PIS in the appropriate foreign language.

Investigator response - In our study settings there might be cross-border patients to be recruited, therefore, the PIS ICF of the XXX language version will be provided to FTM-EC soon after the English version is approved and translated into the XXX language.

Non-approved studies:

FTM-EC rationale - It is not necessary to investigate such interaction in this vulnerable group. If the objective is only to investigate the relationship between this XXX infection and this XXX disease, it can be done in a normal population.

FTM-EC rationale - This type of XXX infected person in Thailand does not have such severe XXX deficiency and thus they are not the targeted population. Even though the study result may show some associations, it will not benefit this type of patient. Besides, some studies in Africa indicated that giving XXX to the patient was worse rather than better.

## Discussion

Ethics committees are responsible for reviewing and deciding whether to approve or not approve research proposals, based on ethical principles, as well as scientific validity. Decisions of ethics committee members are influenced by the variety of backgrounds, perspectives, specialties, experiences, and qualifications of its members. In a study comparing different ethics committees [6], it was reported that even when consensus exists within an ethics committee on the critical issue for approval, interpretations of the decision-making criteria may differ between committees. Multi-center studies across nations, regions, or districts, usually require separate applications to individual committees serving each location. Several of the studies submitted to FTM-EC were multi-center studies; they were required to apply if the study involved FTM personnel or facilities. The decision-making for proposal approval of the FTM-EC is, however, independent and based upon local rules and regulations in the location the study will be performed.

The outcomes of initial review found in this study are similar to studies submitted to IRBs in Africa and South America [13-15], which also reported that 80-90% of protocols submitted for initial review were referred back to investigators for clarification and/or revision. As reported in several other studies regarding the conduct of research among poor and disadvantaged minorities [4,7,9,10,16], the closer examination of “vulnerability” to exploitation, impaired decision-making, or both, are important matters for the ethics committee. However, it was found in this study that the rate of requests for protocol revision after initial review was the same for studies involving minority groups, regardless of extra-vulnerability, at about 90%. The reasons for which proposals were not granted simple approval, and the matters for which clarification and amendment were requested, were similar to other studies [11-15,17], and were mostly related to the informed-consent document and process, and the unclear study design. The rate of deferment/non-approval among studies with and without minority populations at FTM-EC (5-7%) appears not different from ethics committees elsewhere. These rates are similar to the study in Africa (5% not approved) and Brazil (1.7% not approved, 7.4% removed/filed, and 0.5% excluded) [14,15].

Some evidence has been found in the literature [18-20] that tensions between ethics committees and investigators are inevitable and unavoidable, especially when a protocol is deferred or not approved. A universal debate centers on balancing the IRB’s roles--between its responsibility to help protect human subjects and the potential to delay or impede valuable research [17]. However, the FTM-EC has not experienced major conflicts with investigators over its years of operation. According to a qualitative study on the role of the IRB [21], transparency in the review process will help improve perceptions of IRBs among principal investigators. In this regard, at the FTM-EC, all multi-disciplinary aspects of the study are thoroughly discussed and the reasons for deferral and non-approval are explained clearly to the investigators. Moreover, a study on the role of the IRB [21] suggests that tensions could be reduced by educational efforts and shifts in attitudes, including IRB responses to investigator complaints, improvements in relationships, not acting as draconian “ethics police”, but acting as a facilitator and promoter of valuable and ethical research involving human subjects.

The recruitment of participants for research studies is usually challenging, but even more so with vulnerable minorities. Since studies submitted to FTM-EC involving minority groups mostly proposed to recruit displaced populations, cross-border populations, refugees, and legal and illegal migrant workers, for both clinical and non-clinical studies, both panels of the ethics committee focused on vulnerability status. In terms of political status, these people possess fewer defined political rights than people who can claim citizenship within stable national frontiers. Besides, refugee camps themselves give rise to conditions that increase the vulnerability of refuges to potentially abusive research practices. The physical layout of the camps provides some accessibility and a population amenable to systemic sampling and data collection [22]. In addition, they are usually economically impoverished and can be easily influenced to participate in the research at the prospect of minimal financial gain. Among other reasons they are attractive to researchers are the external constraints on movement and location, and the lack of access to healthcare [23]. Follow-up is also easier when study subjects are confined to well-defined geographically isolated areas [7]. However, the “vulnerability” characteristic that is usually considered to be “incapacity or limited capacity of consent” may in fact be a necessary, but not solely sufficient, condition for defining vulnerability status [24]. It has been argued that participation in clinical research provides minority groups with the opportunity to access state-of-the-art medical care, normally unavailable to them; however, a study reviewing the effects of trials [25] suggested that improved outcomes, compared with patients who did not participate in clinical trials, were the results of multiple factors, such as medical intervention, treatment regimen, and extra follow-up care.

While most requests for clarification/revision among all studies related to research methodology (80.7%), the top three for studies involving minority populations were participant information sheet (86.4%), informed consent/assent process (86.4%), and research methodology (84.1%). The rates of request for clarification/revision for studies involving minorities were higher than for all studies in all respects, except for case record form and questionnaire. This concurs with several studies [7,13,22,26], which noted that barriers to informed consent arise from social, cultural, economic, language, educational background, and other factors. In a study of consent among vulnerable populations in Mexico [27], about half of patients found the consent forms difficult to understand; most of the doctors also confirmed that the forms were incomprehensible to patients. In a study on the ethical aspects of tissue research [28], the researchers in many clinical and non-clinical studies tended to describe recruitment and informed consent processes very briefly; this could be resolved by making written detailed guidelines available for investigators, and training researchers to appreciate the sensitive ethical issues surrounding minority groups in research.

At FTM-EC, issues with the recruitment and informed consent process, and problematic or unclear research methodology resulted in both clinical and non-clinical study protocols having to be resubmitted for final approval. The reasons for which research proposals, with and without minority populations, were deferred/non-approved mainly related to scientific merit and research methodology; however, about half concerned the balance of risk-benefit to the individual and community, and violations of human rights. FTM-EC has also deferred/not approved some multi-center studies, especially those enrolling minority and extra-vulnerable populations, which involve sponsors from international agencies or pharmaceutical companies; clear reasons for such review decisions are considered critical. There have also been instances whereby a particular protocol might be approved by certain ethics committees, but not by FTM-EC or by other committees in Thailand. It is thus important to convey the principle that it is the responsibility of the ethics committees in both sponsor and host countries (or central and local levels/sites in multi-center studies) to consider and adhere to scientific and ethical standards, while maintaining their authority not to approve research protocols that fail to meet the standards of the local/host study sites [29]. It is a common practice to trust ethics committees in host countries to play the critical role in protocol review, since they are in a better position to balance the content and context of the protocol against their better understanding of the cultural and moral values of the population where the research is to be conducted [29,30].

On occasion, the FTM-EC recommendation or request for clarification/revision involved the inclusion of community engagement in the proposal. Some case studies of human rights research in low- or middle-income countries have suggested mechanisms for managing conflicts of interest when the research is to be conducted at a specific study location. An effective method is to encourage the use of community engagement and the development of standard ethical operating procedures (SOPs) that will assure the conduct of ethical human research [31]. Studies of the ethical considerations in research being conducted among minority groups suggest the importance of building foundations for community involvement, and establishing trust among eligible participants [32,33]. Linkages with community leaders are essential. They can help to generate a positive attitude towards research activities. Another approach to ensure productive community involvement is the establishment of a community advisory panel. In addition, key informant insiders can share research information consistent with the community’s language and cultural practices [33]. Although FTM-EC does not have a minority representative, the committee contains members with experience working with minorities in remote locations (including cross-border areas and refugee camps).

Moreover, in an attempt to understand minority settings where the research took place and to understand researchers who work in the areas, the FTM-EC has usually performed annual study-site visits in selected remote, rural, border areas. As per its SOP, during these field-site inspections, FTM-EC reviews and examines the research facilities and documentation, and interviews minority research subjects and researchers at the study site. This has facilitated a candid exchange of ideas on the conduct of ethically sound research. Expectations and limitations are openly discussed. This has proved an effective mechanism for creating better understandings and positive relationships between the ethics committee, the investigators in the field, the study subjects, and their communities.

### Limitations of the study

The process and outcomes of proposal review in this study were based on reviews of two ethics committees at one institute in Thailand; the results may reflect those of research involving minority populations in similar settings, but may not be generalizable to other settings. However, the FTM-EC is one of 10 IRBs recognized by Thailand’s Food and Drug Administration and has been reviewing more studies involving vulnerable participants than most other institutes in the country. Its experience can be instructive for other ethics committees dealing with vulnerable participants in very limited resources settings, particularly those with limited education, with migrants, and cross-border populations. The process and outcomes of proposal review by FTM-EC are comparable to other countries, and may serve as a good example of how global standards of research ethics are successfully applied in less-developed regions of the world.

## Conclusions

Like the outcomes of proposal review in other regions, most studies reviewed at FTM-EC required one round of revision and a few were deferred or not approved. Delays in the approval or non-approval of studies involving minority populations were mainly due to inadequate information on the participant information sheet and informed consent/assent form, and unclear research methodology. Other issues on the investigator’s part included inadequate level of awareness, knowledge, and sensitivity among researchers to the vulnerable status of the study participants, and related ethical implications and requirements.

To mitigate problems and avoid unfavorable review outcomes, particularly in studies conducted among vulnerable minority populations, it is recommended that investigators should demonstrate that they have closely considered the necessity of enrolling such populations as well as the benefits of their study to both minority participants and their communities. Like other ethics committees reviewing studies involving minority groups, FTM-EC suggests that investigators should involve participating communities and individuals in the early planning phase, for example, by establishing a community advisory board, whose membership includes the relevant minority groups. Several effective mechanisms that have been demonstrated by the FTM-EC in reducing tensions between investigators and the ethics committee, including transparency in the review process and building good relationships with direct and open communication with the investigators. In addition, FTM-EC has employed capacity-building of ethics committee members and researchers to minimize the need to review problematic issues. Annual FTM-EC refresher training courses for the research community have been conducted over the years; however, this study suggests that it should include sessions to strengthen the capacity and raise awareness of research staff to engage minority groups ethically in productive research.

## Competing interests

The authors declare that they have no competing interests.

## Authors’ contributions

All authors discussed and agreed upon the thematic content, contributed to the development and revision of the draft manuscripts, and read and approved the final version. PA, JK, WW drafted the initial version. KP, SK, WF (FTM-EC Chairpersons) approved the use of information. WC, CL, SP (Ethics Committee Secretariat) extracted unlinked study data from the Ethics Committee archives.

## Authors’ information

PA has been Secretary to FTM-EC for the past 19 years, and is Head of the Office of Research Services. JK is a biostatistics expert, Member of FTM-EC, Assistant Professor in the Department of Tropical Medicine and Hygiene, and Director of the Center of Excellence for Biomedical and Public Health Informatics (BIOPHICS). WW is a current Member and former Member-Secretary of FTM-EC, and an Associate Professor in the Department of Social and Environmental Medicine. KP is a Pediatrician, Chair of FTM-EC Panel I, and Professor in the Department of Tropical Pediatrics. SK is Chair of FTM-EC Panel II, and Professor in the Department of Microbiology and Immunology. WF is a current Expert Member in Medical Social Science, former Chair of FTM-EC, and Head of the Department of Social and Environmental Medicine. WC, CL, and SP, are assistant secretaries to FTM-EC. All are affiliated with the Faculty of Tropical Medicine, Mahidol University.

## Pre-publication history

The pre-publication history for this paper can be accessed here:

http://www.biomedcentral.com/1472-6939/14/33/prepub
